# PAH-associated health risk from ultrafine particles: an urban field study

**DOI:** 10.1007/s11869-026-02118-x

**Published:** 2026-10-12

**Authors:** Wei-Chung Su, Jinho Lee, Masoud Afshar, Kai Zhang, Inkyu Han

**Affiliations:** 1 School of Earth and Environmental Sciences, Baylor University, Waco, TX USA; 2 Department of Environmental Health, School of Public Health, Boston University, Boston, MA USA; 3 Southwest Center for Occupational and Environmental Health, School of Public Health, University of Texas Health Science Center at Houston, Houston, TX USA; 4 Department of Public Health, the University of North Texas Health Science Center at Fort Worth, Fort Worth, TX USA; 5 Department of Epidemiology and Biostatistics, Barnett College of Public Health, Temple University, Philadelphia, PA USA

**Keywords:** Ultrafine particle, Respiratory deposition, PAH, Health risk, Urban area

## Abstract

Ultrafine particles (UFPs) are airborne particulate matter with diameters less than 100 nm. UFPs present in urban atmospheres pose a significant public health issue because they usually carry harmful substances, including toxic metals and polycyclic aromatic hydrocarbons (PAHs). Prolonged exposure to urban UFPs may result in cumulative deposition of these harmful substances in the human respiratory system, elevating the likelihood of adverse respiratory outcomes. Thus, studying UFP respiratory deposition is essential for assessing associated health risks. In this research, a set of field studies was conducted at three selected sampling sites to examine urban UFP exposure in the Greater Houston Area, Texas, USA. At each sampling site, UFP respiratory deposition measurements were performed using the Mobile Aerosol Lung Deposition Apparatus (MALDA). In addition, size-segregated UFP samples were collected and analyzed to identify and quantify harmful constituents associated with UFPs. The resulting data were used to estimate the deposited mass of PAH in human airways and to evaluate the corresponding health risks linked to urban UFP exposure. Overall, both non-carcinogenic and carcinogenic risks associated with UFP-bound PAH in the Greater Houston Area were found to remain within acceptable levels.

## Introduction

The health effects associated with ultrafine particle (UFP) exposure in urban areas have long been a significant public health concern (Schraufnagel 2020). Major sources of UFPs in urban areas include emissions from road and air traffic (Wang et al. 2024), industrial activities (Fujitani et al. 2021), and secondary atmospheric formation (Ahlm et al. 2012). Urban UFPs are mainly generated from fuel combustion and often contain harmful substances such as heavy and transition metals (Utsunomiya et al. 2004), polycyclic aromatic hydrocarbons (PAHs) (Moreno-Ríos et al. 2022), black carbon (Kwon et al. 2020), etc. Due to their extremely small size, UFPs can remain suspended in the ambient air over a prolonged period, allowing them to accumulate to high concentrations in urban environments and elevating the potential for adverse health effects through residents’ exposure (Sioutas et al. 2005). After being inhaled into the airways, a substantial fraction of UFPs will deposit in the lower respiratory tract, particularly within the alveolar region. This deposition facilitates the translocation of harmful substances from UFPs into lung tissue. For instance, exposure to UFPs containing toxic metals and organic compounds has been linked to increased oxidative stress and inflammation in lung cells, which may contribute to a variety of respiratory outcomes (HEI 2013; Ohlwein et al. 2019; Leikauf et al. 2020). Additionally, the entry of harmful substances into the bloodstream may further increase the risk of cardiovascular disease (Samet et al. 2009).

Among the many harmful substances found in UFPs, PAHs represent a major class of concern. PAHs are organic compounds composed of multiple fused benzene rings and primarily formed during the incomplete combustion of fossil fuels (Zhang et al. 2022). The simplest PAH, naphthalene, contains two aromatic rings, whereas larger PAHs consist of five or more rings, such as benzo[a]pyrene. Many PAHs exhibit low vapor pressure, low water solubility, and relatively high melting points (Bojes and Pope 2007). PAHs with higher molecular weight are also chemically stable, making them more resistant to microbial degradation (Haritash and Kaushik 2009; Mojiri et al. 2019). These physicochemical properties promote the persistence of PAHs in airborne particulate matter, including UFPs (Ravindra et al. 2008). It has been reported that PAHs can induce oxidative stress, leading to cellular and tissue damage (Bortey-Sam et al. 2017). As a result, exposure to PAHs has been associated with respiratory and cardiovascular disorders, including asthma and coronary heart disease (Karimi et al. 2015; Mallah et al. 2021). In addition, several PAHs are known to possess carcinogenic and mutagenic properties, which can promote tumor development and initiate DNA damage in exposed populations (Carreras et al. 2013). Table 1 lists selected toxic PAHs, their reference concentrations (* RfC*), unit risks (* UR*), and adverse health effects associated with inhalation exposure. As shown, certain PAHs can cause both noncancer and cancer outcomes (e.g., benzo[a]pyrene and naphthalene), while others pose a risk predominantly for either noncancer or cancer effects.

**Table 1:** Selected PAHs and associated respiratory health effects evaluated in this study: (a) noncancer effects and (b) cancer effects

(a)			
**Substance**	**Noncancer** **effects** ^*^	**RfC** ^*^ **(µg/m** ^**3**^ **)**	**RfD** ^$^ **(mg/kg-day)**
Acenaphthene	Respiratory irritation	2.1E + 2	6.0E-2
Benzo[a]pyrene	Respiratory irritation	2.0E-3	5.7E-7
Benzo[ghi]perylene	Respiratory irritation	7.0E + 0	2.0E-3
Chrysene	-	-	-
Dibenz[a, h]anthracene	Respiratory irritation	-	-
Fluoranthene	Respiratory irritation	1.4E + 2	4.0E-2
Fluorene	Respiratory irritation	1.4E + 2	4.0E-2
Indeno[1,2,3-cd]pyrene	-	-	-
2-Methylnaphthalen	Respiratory effects	1.0E + 1	2.9E-3
Naphthalene	Respiratory effects Nervous effects	3.0E + 0	8.6E-4
Phenanthrene	Respiratory irritation	1.0E-1	2.9E-5
Pyrene	Respiratory effects	1.0E + 2	2.9E-2
(b)			
**S** **ubs** **tance**	**Cancer** **Effects** ^*^ **(Carcinogen Classification)** ^!^	**UR** ^*^ **(µg/m** ^**3**^ **)** ^**−1**^	**CSF** ^$^ **(mg/kg-day)** ^**-1**^
Acenaphthene	- (EPA: -; IARC: 3)^!^	-	-
Benzo[a]pyrene	Lung Cancer (EPA: A; IARC: 1)	6.0E-4	2.1E + 0
Benzo[ghi]perylene	- (EPA: D; IARC: 3)	-	-
Chrysene	Lung Cancer (EPA: B2; IARC: 2B)	6.0E-7	2.1E-3
Dibenz[a, h]anthracene	DNA damage (EPA: B2; IARC 2 A)	6.0E-4	2.1E + 0
Fluoranthene	- (EPA: D; IARC: 3)	-	-
Fluorene	- (EPA: D; IARC: 3)	-	-
Indeno[1,2,3-cd]pyrene	Lung Cancer (EPA: B2; IARC 2B)	6.0E-5	2.1E-1
2-Methylnaphthalen	- (EPA: D; IARC: 3)	-	-
Naphthalene	(EPA: C; IARC 2B)	3.4E-5	1.2E-1
Phenanthrene	- (EPA: D; IARC: 3)	-	-
Pyrene	- (EPA: D; IARC: 3)	-	-

* Sources of RfC, UR, and health effects through inhalation: US EPA Integrated Risk Information System (IRIS), and Michigan Department of Environmental Quality (Chemical Update Worksheet)

^$^ RfD and CSF were converted by RfC and UR, respectively, using the inhalation rate of 20 m3/day and a default body weight of 70 kg (e.g., RfD = RfC × 20/70)

^!^ Cancer classifications: EPA—A (known), B1/B2 (probable), C (possible), D (not classifiable), E (non-carcinogenic); IARC—1 (carcinogenic), 2A (probably carcinogenic), 2B (possibly carcinogenic), 3 (not classifiable), 4 (probably not carcinogenic)

There are numerous studies assessing cancer risks associated with exposure to airborne particle-bound PAHs. Such risk assessments have been conducted in urban and industrial areas worldwide, including Xi’an (China), Okitipupa (Nigeria), Athens (Greece), Tehran (Iran), Hanoi (Vietnam), Algiers (Algeria), Tula (Mexico), Hat Yai (Thailand), and Cyprus (Hoseini et al. 2016; Alves et al. 2017; Teffahi et al. 2021; Trung et al. 2021; Vega et al. 2021; Iakovides et al. 2021; Ediagbonya et al. 2022; Wang et al. 2022; Mahasakpan et al. 2023). However, the majority of these published studies examined PAHs bound with larger aerosol particles, such as PM₂.₅ and PM₁₀. Very few studies have examined PAHs in UFPs and accounted for aerosol respiratory deposition when assessing cancer risks. Therefore, to systematically and comprehensively evaluate the health risks associated with exposure to UFPs in a metropolitan environment, while explicitly accounting for the respiratory deposition fraction of UFPs, our research team conducted a 17-month field study campaign from 2021 to 2022 in the Greater Houston Area. In our field study, the Mobile Aerosol Lung Deposition Apparatus (MALDA) was used to simulate residents’ exposure to urban UFPs to obtain representative size-dependent UFP respiratory deposition data (Su et al. 2021). A commercially available aerosol cascade impactor was also employed to collect size-segregated UFP samples for chemical composition analysis. The overarching goal of this research was to assess the potential health risks associated with exposure to urban UFPs containing toxic metals and PAHs. Because UFP respiratory deposition is size-dependent (ICRP, 1994; NCRP, 1997), integrating size-dependent respiratory deposition measurements obtained using MALDA with size-segregated chemical composition data enables estimation of the respiratory deposited mass of toxic constituents. By accounting for the fraction of inhaled particles that is deposited and retained within the respiratory tract, this approach provides a more physiologically relevant basis for assessing inhalation health risks than conventional inhalation health-risk assessments based solely on ambient pollutant concentrations. In our previously published companion study, health risks attributable to toxic metals in urban UFPs in the Greater Houston Area were evaluated using respiratory deposition data measured by MALDA (Su et al. 2024). The results demonstrated that both non-cancer and cancer risks associated with transition metals in UFPs were below acceptable limits. Building upon that work, the present study applies the same respiratory deposition-based framework to evaluate the health risks associated with PAHs in UFPs. By conducting size-dependent analyses on the two major harmful substances (metals and PAHs) in UFPs, the health risks associated with UFP exposure in the Greater Houston Area can be characterized with greater accuracy.

## Methodology

### Field sampling and measurement

Houston is located in southeast Texas near the Gulf of Mexico and is the fourth most populous city in the United States. The city is intersected by extensive intrastate and interstate highway systems and is surrounded by numerous refineries and chemical facilities, earning it the title “Energy Capital of the World.” Houston is also home to the busy Port of Houston, one of the largest ports in the nation. Houston has a humid subtropical climate, characterized by long, hot summers and mild, cool winters. These factors together make the Greater Houston Area a unique setting for investigating urban UFP exposure from diverse emission sources. Although several studies have examined UFP characteristics around the Greater Houston Area (Phares et al. 2003; Han et al. 2017), respiratory deposition analyses and health risk assessments were not included. In this research, three sampling sites in the Greater Houston Area were selected for field MALDA measurements and UFP sample collections. The characteristics of these three sampling sites were described in detail in our previous publication (Su et al. 2024). Briefly, the Park Place Blvd. (PP) site represents UFP exposure from highway traffic; the Clinton Dr. (CD) site represents UFP exposure from nearby refinery industries, truck exhaust, and the ship channel; and the School of Public Health (SPH) site represents local vehicle exhaust exposure in a hospital district. The field studies were conducted at these three sampling sites sequentially over a 17-month period, covering different seasons.

**Fig. 1:**
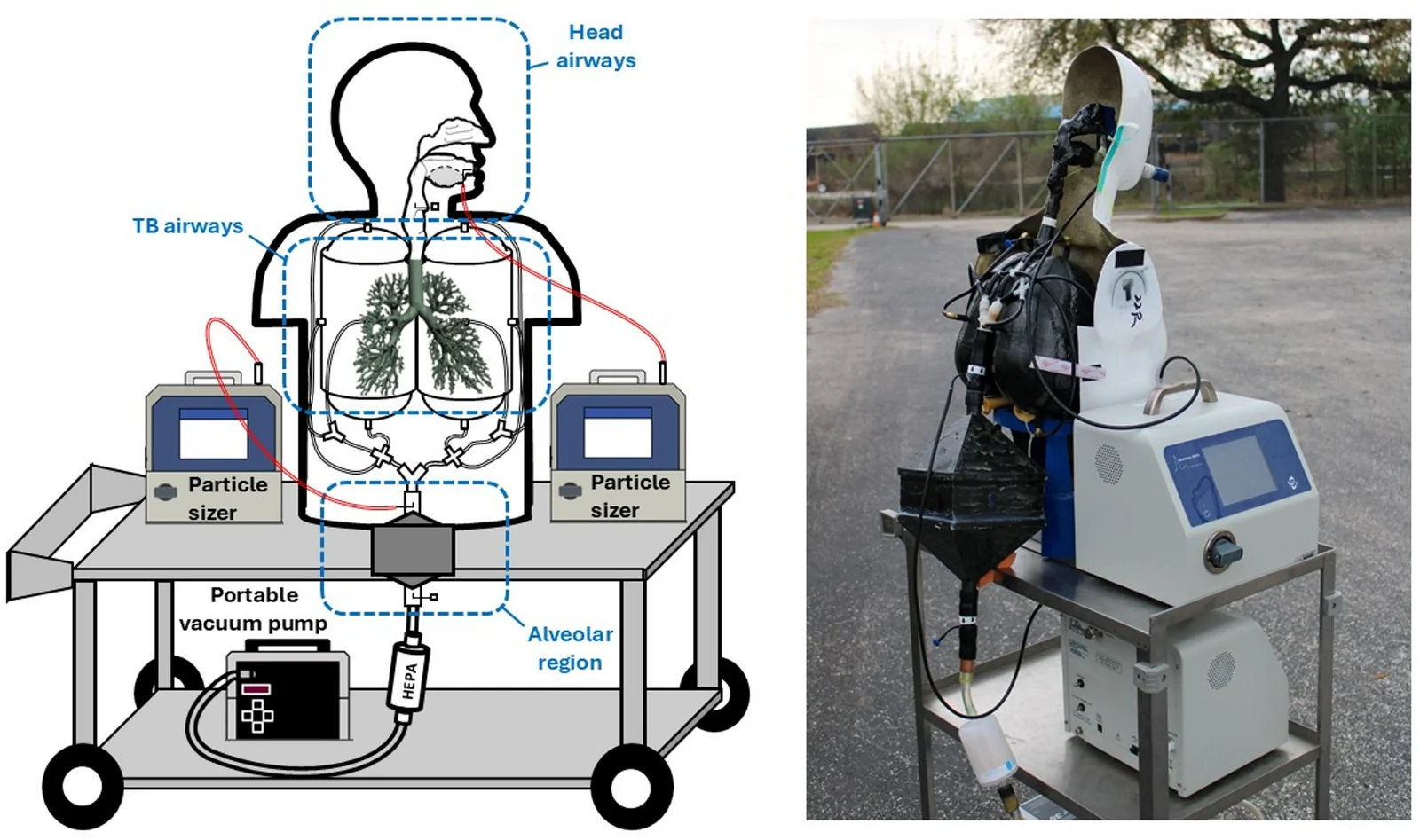
The schematic of the Mobile Aerosol Lung Deposition Apparatus (MALDA) and its application in the field study

MALDA was used to measure on-site, size-dependent UFP respiratory deposition data representing residents’ UFP exposure around the sampling sites. MALDA was operated at an inspiratory flow rate of 30 L/min through the nasal airway, corresponding to a human minute ventilation rate of 15 L/min under symmetric inhalation and exhalation. This ventilation rate is representative of an adult engaged in light outdoor activities, such as walking. Inside the MALDA, there is a set of 3D-printed human airway replicas covering the three major airway regions: the head airways (including the nasal and oral cavities), tracheobronchial (TB) airways (down to the 11th airway bifurcation), and an alveolar region (constructed using conductive porous foam). Comprehensive information on MALDA’s key components and functions is available in our previous publications (Su et al. 2021). Figure 1 shows the schematic and photograph of the MALDA setup at the sampling site. MALDA was deployed at the sampling sites only on days without rain. The principle underlying the use of MALDA to obtain size-dependent aerosol respiratory deposition data is based on measuring the particle size distributions of inhaled UFPs across different airway regions using nanoparticle sizers (NanoScan 3910, TSI Inc., Shoreview, MN, USA). The operating procedures and experimental design used in this field study are detailed in our previous publication (Su et al. 2024). Data obtained by MALDA were utilized to calculate the size-dependent UFP respiratory deposition fractions in the three major airway regions ($\:{DF}_{Head,d}$, $\:{DF}_{TB,d}$, and $\:{DF}_{Alv,d}$).

To collect size-segregated UFP samples at the sampling sites for constituent analysis, the rotating aerosol cascade impactor, MOUDI (110R, TSI Inc., Shoreview, MN, USA), was employed. MOUDI is a multi-stage aerosol cascade impactor capable of collecting particles with aerodynamic diameters ranging from 56 nm to 18 μm across 11 stages. In this research, aerosol samples collected on the final three MOUDI stages (56, 100, and 180 nm) were used to represent UFPs and were analyzed for PAHs. During UFP field sampling, MOUDI was placed at each sampling site for continuous UFP sampling from 8:00 a.m. to 6:00 p.m. (10 h/day) throughout the corresponding measurement period (over one month) to collect cumulative UFP samples to ensure sufficient PAH quantities for subsequent chemical analysis.

### PAHs analysis

Upon completion of UFP sampling, the MOUDI was returned to the laboratory, where the pre-weighed PTFE filters were retrieved, conditioned, and gravimetrically analyzed to determine the collected UFP mass. Each filter was then divided into two portions for metal analysis using ICP/MS (Su et al. 2024) and PAH analysis in the present study. PAHs were extracted following the U.S. EPA TO-13 A method and quantified using a gas chromatography–mass spectrometry (GC/MS) system (Agilent 6890/5975B MSD, Agilent Technologies, Palo Alto, CA). Filters were extracted three times with 30 mL of a dichloromethane–methanol mixture (CH₂Cl₂:CH₃OH, 3:1, v/v) by sonication for 15 min at room temperature. The combined extracts were concentrated to 1 mL and analyzed using the GC/MS equipped with a Restek RXI-5Sil MS capillary column. Samples were injected in splitless mode using a 2-µL programmed temperature vaporization (PTV) injection. Instrument detection limits (IDLs) were determined from five replicate analyses of the lowest calibration standard (5 ng/mL), and the limit of quantification (LOQ) was defined as 3.3 × IDL. Samples with concentrations below the LOQ were excluded from further analysis. The chemical analysis yielded size-dependent PAH-to-UFP mass ratios ($\:{MR}_{k,d}$, where *k* denotes an individual PAH), which were subsequently used to calculate the respiratory deposited mass of each PAH.

### Deposited mass of PAHs

Based on the size-dependent PAH-to-UFP mass ratio obtained from MOUDI analysis, and the on-site, size-dependent UFP respiratory deposition fractions measured by MALDA, the hourly deposited mass of a given PAH (*k*) in the entire respiratory tract, $\:{DM}_{k}$ (mg/hr), can be estimated by:

1 $$\begin{array}{c}DM_k={\sum_d\lbrack\left(DF_{Head,\;d}+DF_{TB,d}+DF_{Alv,d}\right)}\\\times MC_d\times MR_{k,d}\rbrack\times Q\end{array}$$

where $\:{DF}_{Head,d}$, $\:{DF}_{TB,d}$, and $\:{DF}_{Alv,d}$ are the size-dependent UFP respiratory deposition fractions (with values from 0 to 1.0) in the head airways, TB tree, and alveolar region, respectively. $\:{MC}_{d}$ is the size-dependent UFP mass concentration entering MALDA. Based on a published study (Slowik et al. 2004), a UFP density of 1.5 g/cm^3^ was applied according to Levy et al. (2013) to convert the UFP number concentration measured by the particle sizer to the corresponding mass concentration. $\:{MR}_{k,d}$ is the size-dependent PAH-to-UFP mass ratio (mg/mg) of a given PAH (*k*) identified in UFP samples. * Q* is 0.9 (m^3^/hr), representing the total air volume inhaled by an adult per hour during light outdoor activities. It is important to highlight that the $\:{DM}_{k}$ is a size-integrated value summed over the measured particle size distribution.

### Health risk estimation

With $\:{DM}_{k}$ calculated, the daily dose of a given PAH (*k*) in a resident’s body through reasonable urban UFP exposure scenarios can then be estimated:

2 $$\:{ADD}_{k}=\frac{{DM}_{k}\times\:EH\times\:EF\times\:ED\:}{BW\times\:AT}$$

3 $$\:{LADD}_{k}=\frac{{DM}_{k}\times\:EH\times\:EF\times\:ED\:\:}{BW\times\:LT}$$

where $\:{ADD}_{k}$ (mg/kg-day) and $\:{LADD}_{k}$ (mg/kg-day) are the average daily dose and lifetime average daily dose, respectively, of a given PAH (*k*) in a resident’s body. * EH* denotes the estimated daily hours of outdoor UFP exposure for residents proximal to the sampling site, which is set at 3 h/day based on published statistical data (Wu et al. 2010). * EF* represents the exposure frequency, indicating the average number of days per year that residents are exposed to outdoor UFPs, which is assumed to be 350 days/year. * ED* refers to the exposure duration, which is the total number of years a resident is exposed to UFPs. In this research, * ED* is set to 30 years, representing a conventional high-end exposure duration. * BW* is the default human body weight, which is set at 70 kg. * AT* is the averaging time for calculating $\:{ADD}_{k}$, which is equal to the exposure duration (* ED*). * LT* is the lifetime expectancy, which is set at 70 years by default. It should be noted that the values for * EF*, * ED*, * BW*, and * LT* are all based on conventional exposure factors published by the US EPA (1989).

The calculated $\:{ADD}_{k}$ and $\:{LADD}_{k}$ were then used to assess the PAH-induced noncancer and cancer risks, respectively:

4 $$\:Noncancer\:Risk=H{Q}_{k}=AD{D}_{k}/Rf{D}_{k}$$

5 $$\:Cancer\:Risk=LAD{D}_{k}\times\:CS{F}_{k}$$

where $\:{HQ}_{k}$ is the noncancer hazard quotient for a given PAH (*k*). $\:{RfD}_{k}$ (mg/kg-day) is the noncancer reference dose for the PAH (*k*). $\:{CSF}_{k}$ (kg-day/mg) is the cancer slope factor of the PAH (*k*). The required $\:{RfD}_{k}$ and $\:{CSF}_{k}$ values for conducting PAH risk assessments in this research are listed in Table 1. For noncancer risk assessment, a calculated $\:{HQ}_{k}$ greater than 1.0 indicates a potentially unacceptable noncancer health risk. The hazard index (* HI*) was applied to all PAHs that can induce the same noncancer effects and is calculated as * HI* =∑ $\:{HQ}_{k}$, the sum of all associated $\:{HQ}_{k}$ values. When the calculated * HI* exceeds 1.0, the noncancer health risk is also considered unacceptable. For cancer risk assessment, if the estimated lifetime excess cancer risk exceeds 1 × 10^− 6^ (one in one million), the risk is considered unacceptable.

## Results

Based on the field study design, measurements at the PP site were conducted during two periods: May–July 2021 (hot season) and January–March 2022 (cool season). In total, 39 MALDA datasets were collected at this site, along with one MOUDI dataset during each period. At the CD site, sampling occurred during August–October 2021 (hot) and April–May 2022 (cool). These campaigns yielded 63 MALDA datasets in total, with one MOUDI dataset collected during each period. At the SPH site, field measurements were conducted in October–December 2021 (cool) and August–September 2022 (hot). A total of 62 MALDA datasets were obtained, accompanied by one MOUDI dataset per sampling period.

**Fig. 2:**
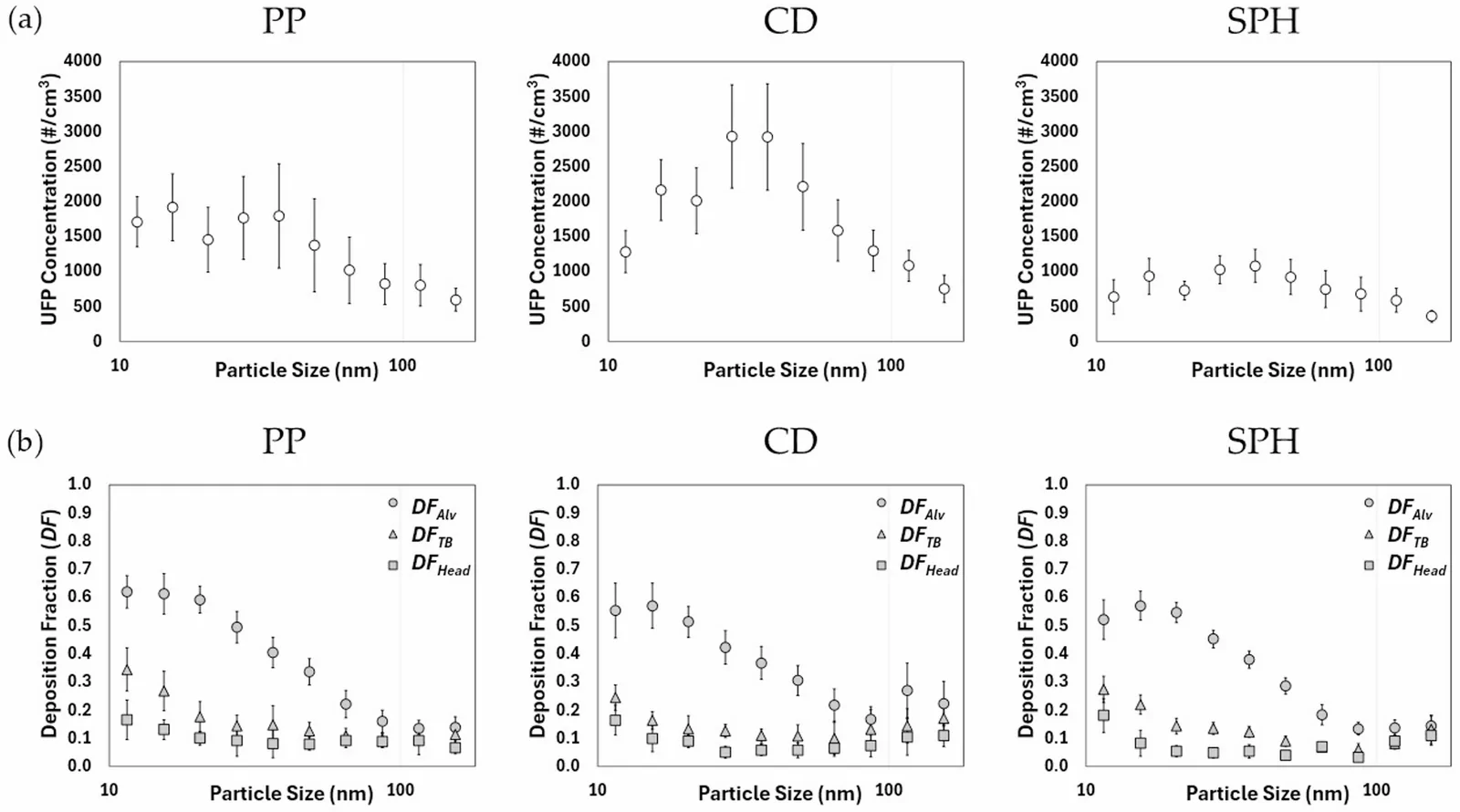
(**a**) Particle size distributions of UFPs at each sampling site, and (**b**) size-dependent UFP respiratory deposition fractions ($\:{DF}_{Head,d}$, $\:{DF}_{TB,d}$, and $\:{DF}_{Alv,d}$) measured by MALDA. PP = Park Place Blvd.; CD = Clinton Dr.; SPH = School of Public Health

Figure 2 shows the particle size distributions of UFPs measured at different sampling sites and the associated UFP respiratory deposition fractions obtained by MALDA. The particle size distributions (Fig. 2a) were measured by the particle sizer connected to the MALDA inlet, representing the UFPs inhaled into the human airways. The particle size distribution is presented as the average of all data collected at each sampling site, covering three sampling time frames during the day (morning rush hour, noon, and afternoon rush hour) and two seasons (hot and cool). As can be seen, the measured peak diameters of UFPs were approximately 40 nm at all sampling sites. The CD site (industrial area) exhibits a relatively higher average UFP concentration among the three sampling sites, whereas the SPH site (hospital district) exhibits a relatively lower UFP concentration. Figure 2b presents the size-dependent UFP respiratory deposition fractions in the three major airway regions ($\:{DF}_{Head,d}$, $\:{DF}_{TB,d}$, and $\:{DF}_{Alv,d}$). According to Eq. (1), the size-dependent respiratory deposition fractions play a key role in estimating the deposited mass of PAHs in human airways. The deposition fractions shown in Fig. 2b represent the average of all data acquired at each sampling site across different sampling periods. As shown, the alveolar region exhibited a relatively higher UFP deposition fraction than the head and TB airways, with the highest deposition fraction observed at 15 nm. The patterns of UFP respiratory deposition fractions were comparable among the three sampling sites. This is a reasonable result because it is known that aerosol respiratory deposition fraction is primarily a function of particle size (Hinds and Zhu 2022). Based on this, under similar respiratory deposition fractions, a higher ambient UFP concentration will result in a greater amount of UFPs deposited in the airways. Increased UFP respiratory deposition will introduce a greater mass of PAHs into the lung, leading to higher health risks.

Table 2 lists the size-dependent PAH-to-UFP mass ratios, $\:{MR}_{k,d}$, for a given PAH (*k*) detected in the UFP samples. The $\:{MR}_{k,d}$ is another essential experimental parameter used in Eq. (1) to estimate the deposited mass of a given PAH (*k*) in the human airways ($\:{DM}_{k}$). These $\:{MR}_{k,d}$ values were obtained from gravimetric and chemical analyses performed on size-segregated UFP samples collected using MOUDI. It can be seen that the value of $\:{MR}_{k,d}$ are generally below the order of 10^− 4^, with most falling within the 10^− 6^ range. Several PAHs exhibit a trend of increasing $\:{MR}_{k,d}$ as UFP particle size decreases, suggesting that volatile components of UFPs are gradually lost, leaving the less volatile PAHs enriched in smaller particles. The data shown in Table 2 represents the average of two samples collected at each sampling site from two different seasons. The limited number of samples primarily resulted from the inherent difficulty of collecting size-segregated UFP samples with a MOUDI while obtaining sufficient particulate mass to meet the detection limits required for both metal and PAH analyses. Because the MOUDI separates aerosol into multiple size fractions, only small amounts of particles are collected in each fraction. Consequently, accumulating sufficient particulate mass in the UFP size range (particularly for particles around 56 nm in diameter) for both chemical analyses requires approximately 300–400 h of cumulative sampling (10 h per day over 30–40 sampling days). Preliminary evaluations showed that samples collected over a single day, or even one week, did not yield sufficient particulate mass for reliable quantification. Accordingly, the two UFP samples are cumulative, time-integrated composite samples collected during different seasons that provide estimates of the average metal and PAH composition in UFPs at each sampling site.

**Table 2:** Size-dependent PAH-to-UFP mass ratio ($\:{MR}_{k,d}$) at different sampling sites. PP = Park Place Blvd.; CD = Clinton Dr.; SPH = School of public health; d represents particle diameter (nm)

	PP			CD			SPH		
	56 < d < 100	100 < d < 180	180 < d < 320	56 < d < 100	100 < d < 180	180 < d < 320	56 < d < 100	100 < d < 180	180 < d < 320
Acenaphthene	1.0E-04	3.5E-05	1.3E-05	1.7E-05	8.0E-06	3.3E-06	4.2E-05	2.1E-05	8.7E-06
Benzo[a]pyrene	-	-	2.0E-06	1.7E-06	2.4E-06	1.3E-06	-	-	1.7E-06
Benzo[ghi]perylene	5.3E-06	3.6E-06	3.2E-06	7.0E-09	1.8E-06	2.2E-06	4.6E-06	5.6E-06	3.9E-06
Chrysene	1.0E-04	3.5E-05	1.3E-05	1.7E-05	8.0E-06	3.3E-06	4.2E-05	2.1E-05	8.7E-06
Dibenz[a, h]anthracene	-	-	2.2E-07	1.9E-06	-	-	-	4.0E-06	-
Fluoranthene	-	1.1E-06	3.0E-07	3.1E-06	3.1E-06	1.6E-06	1.2E-07	2.1E-06	1.3E-06
Fluorene	-	-	-	1.8E-09	7.0E-07	3.6E-07	-	-	-
Indeno{1,2,3,cd}pyrene	-	3.0E-06	2.6E-06	-	3.3E-09	1.2E-06	1.5E-06	4.2E-06	3.4E-06
2-methyl-Naphthalen	6.5E-06	2.9E-06	2.0E-07	-	-	-	2.1E-05	7.0E-06	-
Naphthalene	4.6E-05	2.4E-05	4.7E-06	1.4E-05	9.5E-06	3.5E-06	7.1E-05	2.1E-05	4.3E-06
Phenanthrene	4.8E-06	3.1E-06	7.4E-07	4.5E-06	4.1E-06	2.3E-06	7.4E-06	4.3E-06	2.1E-06
Pyrene	-	5.5E-07	-	2.4E-06	2.4E-06	1.2E-06	1.7E-07	1.9E-06	1.1E-06

Based on the size-dependent UFP measurement data and respiratory deposition data obtained using MALDA (Fig. 1), together with the PAH mass fraction data acquired from the MOUDI chemical analysis (Table 2), the size-integrated hourly deposited mass of a given PAH ($\:D{M}_{k}$) in the human airways could be estimated according to Eq. (1). Table 3 lists the calculated $\:D{M}_{k}$ for the PAHs detected in this research. As can be seen, the $\:D{M}_{k}$ values span a wide range (approximately 10^− 10^ to 10^− 8^ mg/hr) across the sampling sites, primarily attributable to differences in PAH mass fractions in UFPs. Moreover, the $\:D{M}_{k}$ was generally lower at the SPH site than at the PP and CD sites, which corresponds to the relatively low ambient UFP concentrations measured at SPH in the hospital district.

**Table 3:** The estimated hourly deposited mass (mg/hr) of a given PAH ($\:{DM}_{k}$) in the airways for different sampling sites. PP = Park place blvd.; CD = Clinton Dr.; SPH = School of public health

	PP	CD	SPH
Acenaphthene	5.2E-08	1.7E-08	1.7E-08
Benzo[a]pyrene	1.0E-09	3.6E-09	7.3E-10
Benzo[ghi]perylene	4.5E-09	3.2E-09	3.9E-09
Chrysene	5.2E-08	1.7E-08	1.7E-08
Dibenz[a, h]anthracene	1.2E-10	9.6E-10	9.1E-10
Fluoranthene	4.9E-10	5.0E-09	1.1E-09
Fluorene	-	7.8E-10	-
Indeno{1,2,3,cd}pyrene	2.3E-09	1.2E-09	2.7E-09
2-methyl-Naphthalen	3.2E-09	-	5.8E-08
Naphthalene	2.5E-08	1.6E-08	2.1E-08
Phenanthrene	2.9E-09	7.0E-09	3.4E-09
Pyrene	1.6E-10	3.8E-09	9.5E-10

Table 4 summarizes the health risks associated with exposure to urban UFPs containing PAHs. These risks were obtained based on the exposure scenario involving 3 h of daily outdoor activity. The noncancer risks were calculated using Eqs. (2) and (4), while the cancer risks were determined using Eqs. (3) and (5). The required $\:{RfD}_{k}$ and $\:{CSF}_{k}$ values used in the risk assessment were sourced from Table 1. Table 4a shows that all calculated * HQ* values are less than 1.0, indicating the noncancer risks associated with exposure to UFPs containing PAHs in the Greater Houston Area are within an acceptable range. In addition, because all PAHs listed in Table 1 induce similar noncancer respiratory effects (e.g., respiratory irritation or other respiratory impacts), the corresponding * HQ* values in Table 4a may be summed to obtain the * HI* for evaluating the overall noncancer risk. The resulting * HI* values were found to be in the order of 10^− 5^ to 10^− 4^, suggesting that PAH-induced respiratory harm is not expected to be significant. Table 4b shows that the estimated cancer risks associated with PAHs also fall within the acceptable range. The calculated excess cancer risks ranged from 10^− 13^ to 10^− 11^, which is far below the conventional acceptable cancer risk level of 10^− 6^. Therefore, based on the health risk results obtained in this research, community exposure to urban UFPs in the Greater Houston Area is unlikely to result in PAH-related health effects.

**Table 4:** Estimated health risks associated with exposure to urban UFPs containing PAHs for communities in the greater houston area (a) noncancer risks (*HQ* and *HI*), and (b) cancer risk. PP = Park place blvd.; CD = Clinton Dr.; SPH = School of public health

(a)			
	**PP**	**CD**	**SPH**
Acenaphthene	3.6E-08	1.2E-08	1.2E-08
Benzo[a]pyrene	7.4E-05	2.6E-04	5.3E-05
Benzo[ghi]perylene	9.3E-08	6.6E-08	7.9E-08
Fluoranthene	5.0E-10	5.1E-09	1.1E-09
Fluorene	-	8.0E-10	-
2-methyl-Naphthalen	4.5E-08	-	8.2E-08
Naphthalene	1.2E-06	7.9E-07	1.0E-06
Phenanthrene	4.1E-06	1.0E-05	4.8E-06
Pyrene	2.3E-10	5.4E-09	1.3E-09
HI	8.0E-05	2.7E-04	5.9E-05
(b)			
	**PP**	**CD**	**SPH**
Benzo[a]pyrene	3.8E-11	1.3E-10	2.7E-11
Chrysene	1.9E-12	6.3E-13	6.3E-13
Dibenz[a, h]anthracene	4.3E-12	3.5E-11	3.4E-11
Indeno{1,2,3,cd}pyrene	8.3E-12	4.4E-12	1.0E-11
Naphthalene	5.3E-11	3.5E-11	4.5E-11

## Discussion

In our previous study on toxic metals in urban UFPs (Su et al. 2024), UFP size distribution and respiratory deposition were evaluated across multiple daily time periods and seasons, which introduced substantial variability and complexity in data analysis and presentation. In the present PAH-focused study, these parameters are therefore presented as overall site-averaged values across all sampling periods to reduce redundancy and simplify the calculation of $\:D{M}_{k}$. This averaging approach also provides a clearer representation of UFP size distribution and respiratory deposition patterns (Fig. 2). Similarly, PAH-to-UFP mass ratios ($\:D{M}_{k}$) were reported as annual averages (Table 2) for the purpose of consistency in this research. Assessing health risks using annual average data rather than season-specific data is unlikely to affect the final results substantially. Instead, using annual averages provides a more concise dataset and highlights the key features of the data more clearly. Russell et al. (2004) reported that, on seasonal and annual averaging timescales, the mass concentrations and chemical compositions of PM₂.₅, which includes the ultrafine particle (UFP) size range, were generally homogeneous across Southeast Texas, including the Greater Houston Area. Although UFPs may exhibit greater short-term temporal variability than the larger PM₂.₅ fraction, these findings support the use of time-integrated measurements for estimating long-term average particulate exposure in the Greater Houston Area. Although time-averaged concentrations do not fully resolve short-term fluctuations associated with daily traffic patterns or episodic industrial emissions, the extended MOUDI sampling period (> 30 days) produced time-integrated composite samples that incorporate temporal variability into cumulative measurements. Moreover, while short-term UFP concentration peaks may briefly exceed long-term average concentrations, their contribution to the estimated chronic average daily PAH dose and the resulting lifetime health-risk estimates is expected to be limited because exposure is averaged over a 30-year period.

As exhibited in Fig. 2, the size-dependent UFP respiratory deposition fractions obtained at the three sampling sites demonstrated similar patterns. In addition, as presented in Table 2, no site showed a particularly elevated PAH-to-UFP mass ratio. Based on these data acquired, the concentration of UFPs (i.e., particle size distribution) is the key factor determining the deposited mass of a given PAH in the lung ($\:{DM}_{k}$). Lower UFP concentrations lead to reduced UFP respiratory deposited mass, resulting in lower PAH intake in the airways. Therefore, residents living near the SPH sampling site in the Texas Medical Center area (primarily apartment complexes) have relatively lower PAH intake due to the low UFP concentrations in the hospital district (Fig. 2a). In contrast, residents living near the PP and CD sampling sites have increased PAH intake due to higher local UFP concentrations generated by traffic or industrial activities. A greater PAH deposited mass may lead to higher PAH daily doses and elevated risks of PAH-induced health effects. Nevertheless, the estimated health risks associated with PAHs at all sampling sites were found to be within acceptable levels in this research. This outcome may be attributed to the low PAH-to-UFP mass ratios ($\:{MR}_{k,d}$) observed.

The use of standard U.S. EPA exposure assumptions is common in health-risk assessments of the general population because it provides a consistent basis for comparing results across studies. However, actual exposure factors, including time spent outdoors, inhalation rate, and body weight, may vary considerably among individuals and subpopulation groups. Because inhalation dose is directly proportional to exposure hours and inhalation rate and inversely proportional to body weight, individuals with longer outdoor exposure times, higher breathing rates, or lower body weights may experience higher inhalation doses than those estimated in this study. For example, children’s lower body weight may result in inhalation doses on a body-weight basis that are approximately 2–3 times greater than those of adults, despite their lower inhalation volumes. Outdoor workers typically spend longer periods outdoors (8 h/day) and, during moderate-to-heavy work, have inhalation rates 50% higher than the breathing rate assumed in this study. Consequently, their inhalation doses and associated health risks could be up to four times greater than those estimated for the general population. Nevertheless, because the estimated noncancer and cancer risks in this study are several orders of magnitude below their acceptable risk levels, reasonable variations in exposure assumptions are not expected to alter the overall conclusion that the estimated health risks remain within acceptable levels, including vulnerable populations and populations with greater exposure.

Among the exposure factors used in Eqs. (2) and (3) to calculate $\:{ADD}_{k}$ and $\:{LADD}_{k}$ for the noncancer and cancer risk assessments, deposited mass ($\:{DM}_{k}$) and exposure hours (* EH*) were identified as the primary sources of uncertainty. To quantify the uncertainty in the health risk assessment, a probabilistic Monte Carlo analysis (5,000 iterations) was performed using the probability distributions of $\:{DM}_{k}$ and the published distribution of * EH* (Wang et al. 2022). Because $\:{DM}_{k}$ is calculated from both the respiratory deposition fractions and the PAH-to-UFP mass ratio, the uncertainties associated with these parameters were propagated through the calculation of $\:{DM}_{k}$, and were inherently incorporated into the Monte Carlo analysis. The results of the highest estimated 95th-percentile noncancer risk was 2.3 × 10^− 3^ for benzo[a]pyrene at the Clinton Dr. (CD) site, while the highest estimated 95th-percentile cancer risk was 9.0 × 10^− 9^ for dibenz[a, h]anthracene at the Park Place Blvd. (PP) site. For all other PAHs, the estimated 95th-percentile noncancer risks ranged from 8.5 × 10^− 9^ (fluorene at CD) to 8.7 × 10^− 5^ (phenanthrene at CD), and cancer risks ranged from 9.0 × 10^− 12^ (chrysene at PP) to 1.2 × 10^− 9^ (benzo[a]pyrene at CD). All estimated 95th-percentile noncancer and cancer risks remained well below their respective acceptable thresholds, indicating that exposure to urban UFP-bound PAHs in the Greater Houston Area is associated with acceptable health risks despite the identified uncertainties.

As mentioned in the Introduction, many studies worldwide have evaluated cancer risks associated with exposure to particle-bound PAHs in ambient air. Reported lifetime cancer risks span a wide range, from approximately 10⁻⁷ in Athens (Greece) and Cyprus (Alves et al. 2017; Iakovides et al. 2021), to the 10⁻⁶–10⁻⁵ range in Algiers (Algeria), Xi’an (China), Okitipupa (Nigeria), and Tehran (Iran) (Hoseini et al. 2016; Teffahi et al. 2021; Ediagbonya et al. 2022; Wang et al. 2022), and reaching the unacceptable 10⁻⁴ level in Hanoi (Vietnam) and Tula (Mexico) (Trung et al. 2021; Vega et al. 2021). Based on the published data summarized above, most estimated cancer risk values are several orders of magnitude higher than those obtained in this study. However, these risk assessments were primarily based on PM₂.₅ or PM₁₀ measurements, which involve particles with substantially larger diameters and mass than UFPs (particle mass scales with d³). Consequently, larger particles can carry greater amounts of PAHs. Only one study reported separate cancer risk assessments for PM₀.₁, PM₁, and PM₂.₅ (Mahasakpan et al. 2023). In that study, the estimated cancer risk for PM₀.₁ (UFPs) ranged from 10⁻⁹ to 10⁻⁸, which is of the same order of magnitude as the values obtained in the present research. Nevertheless, none of these studies accounted for aerosol respiratory deposition fractions. All inhaled particles and their bound PAHs were implicitly assumed to deposit entirely within the respiratory tract and contribute to the estimated cancer risk.

As also previously noted, the major toxic constituents of urban UFPs are metals, PAHs, and black carbon, with black carbon serving as a carrier for both PAHs and metals. Thus, the overall health risk associated with urban UFP exposure should comprise the individual risks posed by these harmful substances. However, the risk assessment for black carbon in UFPs could not be performed because no conventional $\:{RfD}_{k}$ or $\:{CSF}_{k}$ values for black carbon are published by government agencies such as the EPA and ATSDR. As a result, in this research, the integrated health risk associated with urban UFP exposure can only be assessed using the available data on PAHs and toxic metals. Table 5 summarizes the health risks caused by UFP exposure in the Greater Houston Area. The data presented combine the metal-related health risks reported in our previous publication with the PAH-related health risks obtained in the current study. It is worth noting that, because the inhalation-induced noncancer effects caused by toxic metals and PAHs are predominantly respiratory outcomes, the noncancer risks are expressed as the hazard index (* HI*), which represents the sum of individual * HQ* values. As can be seen in Table 5, all estimated health risks fall within acceptable levels, indicating that urban exposure to UFPs in the Greater Houston Area is generally within a safe range. The noncancer * HI* values were several orders of magnitude below the value of 1.0, and the estimated cancer risks were also below the conventional acceptable risk level of 10^− 6^ (one in one million). Notably, both noncancer and cancer risks associated with toxic metals were consistently higher than those associated with PAHs.

**Table 5:** Summary of health risks associated with exposure to toxic metals and PAHs in urban UFPs from the greater houston area. PP = Park place blvd.; CD = Clinton Dr.; SPH = School of public health

		PP/CD/SPH
**Noncancer**	Metal ^#^	3.5E-03–3.8E-03
	PAH ^#^	5.9E-05–2.7E-04
**Cancer**	Cr	9.7E-08–2.7E-07
	Pb	5.4E-11–2.1E-10
	Ni	1.2E-09–7.3E-09
	As	1.8E-08–4.7E-08
	Cd	5.0E-09–2.0E-08
	Benzo[a]pyrene	2.7E-11–1.3E-10
	Chrysene	6.3E-13–1.9E-12
	Dibenz[a, h]anthracene	4.3E-12–3.5E-11
	Indeno{1,2,3,cd}pyrene	4.4E-12–1.0E-11
	Naphthalene	3.5E-11–5.3E-11

^#^ Hazard Index (HI), obtained by summing all HQs with similar respiratory effects

While the estimated noncancer and cancer risks in this study were within acceptable levels, the assessment was limited to the parent PAHs analyzed and their associated respiratory health risks. Therefore, the results should not be interpreted as representing the overall health burden associated with exposure to UFPs or even the complete health risks associated with PAHs. Urban UFPs contain numerous other potentially toxic constituents, including oxygenated PAHs, nitrated PAHs, alkylated PAHs, black carbon, and hazardous organic compounds, many of which have been associated with oxidative stress, inflammation, and adverse respiratory effects. These toxic constituents were beyond the scope of the current study and were not included in the health-risk assessment. Consequently, the estimated risks in this research represent only a portion of the overall health burden attributable to ambient UFP exposure. Future studies incorporating a broader range of UFP-associated toxic constituents and PAH derivatives are needed to provide a more comprehensive evaluation of the health risks associated with urban UFP exposure. An improved understanding of UFP-associated health risks will enable policymakers to develop more effective mitigation strategies, including stricter industrial regulations and more stringent vehicle emission standards. In addition, communities can receive evidence-based public health recommendations, such as air quality advisories and guidance for limiting outdoor activities during poor air quality episodes, thereby helping communities reduce UFP exposure and protect public health.

## Conclusions

This research employed a unique field-based experimental approach that integrates size-dependent UFP respiratory deposition measurements obtained using MALDA with size-segregated chemical composition analysis using MOUDI to quantify the respiratory deposited mass of PAHs. Unlike conventional inhalation health-risk assessments based solely on ambient pollutant concentrations, this approach accounts for the fraction of inhaled particles that is deposited and retained within the respiratory tract, thereby providing a more physiologically relevant basis for assessing inhalation health risks. The results indicate that health risks associated with urban UFP-bound PAHs are governed primarily by ambient UFP concentrations and the PAH mass fractions within UFPs. Overall, the estimated noncancer and cancer risks associated with the subset of parent PAHs examined in this study were within acceptable levels for the Greater Houston Area under the evaluated exposure scenarios. This deposited mass-based framework offers a transferable experimental methodology for assessing inhalation health risks from UFPs and other nanoparticles across a wide range of environmental and occupational exposure settings. 

## Data Availability

The datasets generated and/or analyzed during the current study are available from the corresponding author upon reasonable request.

## Declarations

### Use of artificial intelligence

AI-assisted tools were used in language editing and grammar check when drafting this manuscript.
